# Utility and outcomes of routine jejunostomy placement following oesophagectomy

**DOI:** 10.1308/rcsann.2025.0045

**Published:** 2025-08-15

**Authors:** H TW Chon, A Botros, I El-Zayat, SJ Mercer, J Straatman, G van Boxel, PH Pucher

**Affiliations:** ^1^ Portsmouth Hospitals University NHS Trust, UK; ^2^ University of Portsmouth, UK

**Keywords:** Jejunostomy, Oesophagectomy, Enteral feeding

## Abstract

**Introduction:**

Feeding jejunostomy (FJ) is used widely as a nutritional adjunct in oesophagectomy. However, FJ placement is also associated with additional morbidity. While FJ may be invaluable in some patients, particularly in those who suffer postoperative complications, it may be an avoidable source of distress and morbidity in others. This study aimed to assess the utility and outcomes of FJ in patients with uncomplicated recovery after oesophagectomy.

**Methods:**

Outcomes for 100 consecutive patients who underwent oesophagectomy with uncomplicated recovery (Clavien-Dindo ≤2) were included from a prospectively maintained database. All had routine FJ placement. Demographic, disease, operative and clinical outcomes were analysed. Necessity of FJ usage (as assessed by specialist dietician) and complications were recorded. Differences between patients requiring postdischarge FJ use, and those who did not, were assessed.

**Results:**

Complete data for a total of 97 patients were included. Overall, only 9/97(9.3%) patients required ongoing FJ usage on discharge. No significant differences in demographics between two groups were observed. Postoperative complications were seen in 42/97(43.3%) patients, most commonly respiratory complications. FJ-related complications were recorded in 18/97(18.5%) patients, most commonly jejunostomy tube displacement.

**Conclusions:**

The low rate of postoperative FJ usage and relatively high risk of associated complications suggests that a selective FJ placement strategy may have positive implications for patients over a routine placement strategy. No significant predictive factors for requiring postoperative FJ use were identified; work to define optimal feeding adjunct strategies in the postoperative context is needed.

## Introduction

Patients with oesophageal cancer often experience significant weight loss and are at high risk of malnutrition due to direct effects of the disease, as well as side-effects of surgical or oncological therapy.^1,2^ Among those patients undergoing radical treatment, preoperative weight loss and malnutrition have been demonstrated to have a negative impact on overall survival after oesophagectomy.^3,4^ Preoperative malnutrition is often associated with unfavourable long-term outcomes after oesophagectomy and it is an independent factor in survival prediction.^5^ Similarly, postoperative malnutrition is common and occurs in more than 50% of patients after oesophagectomy.^6^ Patients who experienced malnutrition after surgery can have prolonged recovery time and lower quality of life.^7^ They are also less likely to tolerate adjuvant chemotherapy, which can further affect long-term survival.^8,9^ Specialist dietetic input, and ensuring adequate nutrition and caloric intake postoesophagectomy is therefore a key component of patient care in the management of oesophageal cancer.^10^

Feeding jejunostomy (FJ) is a nutritional adjunct used commonly in oesophagectomy and is sited at time of surgery for use in the postoperative phase by some centres.^11^ The usage of FJ may vary from routine immediate usage postoperatively, including patient discharge with FJ supplementation at home, to the placement of FJ as an emergency adjunct to be used only in cases where patients are unable to achieve sufficient oral intake.^12^ However, FJ placement is also associated with potential for additional morbidity. The reported incidence of minor complications such as gastrointestinal complaints like nausea, diarrhoea and abdominal distension, jejunostomy site leakage and FJ dislocation ranges from 0.7% to 39%.^13,14^ Incidence of serious complications, including bowel obstruction, jejunostomy tube-related obstruction, chronic jejunocutaneous fistula and jejunostomy site infection are reported to range between 2% and 15%.^8,14^ Nonocclusive small bowel ischaemia – a scarce but significant complication related to FJ – has an incident rate of 1.8% and a high mortality rate of 65.2%.^15^ Additionally, patients’ opinions and experiences towards FJ have been negative overall, with reasons being discomfort and pain caused by stitches and the jejunostomy tube, gastrointestinal complaints and disturbed sleep pattern.^16^

The clinical usage of FJ in the context of oesophagectomy is, therefore, variable. Proponents cite the nutritional benefits and potential to improve ability to complete multimodal therapy, whereas detractors cite the additional morbidity, particularly in patients who manage sufficient oral intake and therefore derive no benefit from FJ placement. A recent report from the UK National Oesophago-Gastric Cancer Audit (NOGCA) highlighted the significant variation in postoesophagectomy nutritional approach in the UK. While the most common approach remains to place FJ, routine postoperative feeding strategies range from oral alone, to FJ, to naso-enteral to parenteral nutrition.^17^

With improving outcomes and more focus on enhanced recovery principles including specialist nutritional and dietetic care, we wished to explore the necessity of FJ in all patients after oesophagectomy.^18^ As treatments become increasingly personalised, it may be that patients benefit from a selective, rather than universal, strategy of FJ placement. One rationale is that, particularly in the era of minimally invasive oesophagectomy with a reduced risk of adhesions, the minority of patients who suffer major complications (these being the patients most likely to require long-term feeding adjuncts) could easily have FJ placed at the time of return to the operating theatre (or any time FJ might become necessary). We sought therefore to assess the utility and outcomes of FJ in patients with uncomplicated recovery after oesophagectomy.

## Methods

This was a single-centre retrospective analysis at Portsmouth Hospitals University NHS Trust, Portsmouth, UK. This analysis was permitted as a service evaluation following local institutional review. Outcomes for 100 consecutive patients who underwent oesophagectomy with uncomplicated recovery (minor complications, defined as Clavien-Dindo ≤2), between September 2020 to November 2023 inclusive who had FJ placed at time of surgery, were extracted from a prospectively maintained database. Patients were excluded from the study if they did not have FJ placed at time of oesophagectomy (including if they had one placed preoperatively, for example, at staging laparoscopy), required other feeding adjunct including nasogastric or nasojejunal feeding preoperatively (as these patients could be reasonably expected to require postoperative supplementation and would therefore be unlikely to be considered for surgery without FJ placement), required reintervention under general anaesthetic in the postoperative phase (major complication) or suffered in-hospital death.

### FJ technique and use

At time of data collection, practice in our centre was to routinely place FJ at time of oesophagectomy in all patients. This was done in a standardised minimally invasive fashion. The ligament of Treitz was identified and a suitable segment of proximal jejunum approximately 30cm distal secured circumferentially around a Freka enteral feeding tube (Fresenius Kabi) to the abdominal wall in the left upper quadrant using sutures. Enteral feed supplementation via FJ was routinely administered postoperatively in a standardised regimen up to a target volume set by a specialist dietician. Postoperative care was carried out in accordance with a standardised enhanced recovery protocol. Nutritional assessment was performed by a specialist oesophagogastric dietician throughout the care pathway. Those requiring ongoing nutritional supplementation on discharge continued jejunostomy feeding according to a preset regimen subject to routine outpatient dietetic review. For patients able to maintain adequate oral nutrition on discharge, FJ was left in situ on discharge and flushed daily with saline to maintain patency. FJ was then removed at 6–8 weeks postinsertion during outpatient clinic review if not required to maintain adequate nutrition at that point.

### Data collection and analysis

Relevant data were collected including demographic and disease variables: age, sex, comorbidities, TNM stage, body mass index (BMI) at the time of diagnosis and at time of oesophagectomy, malnutrition universal screening tool (MUST) score at the time of diagnosis, operative and clinical outcomes. Individual details of those patients requiring FJ feeding at discharge were assessed.

Data were compared between groups of patients who continued with FJ feeding upon discharge, and those who did not. Appropriate nonparametric tests were used (Chi-square and Mann–Whitney *U* test); *p* values of <0.05 were considered significant. Data were collated in Microsoft Excel (Microsoft Corp). Analysis was performed in SPSS (IBM Corp).

## Results

**Figure 1:**
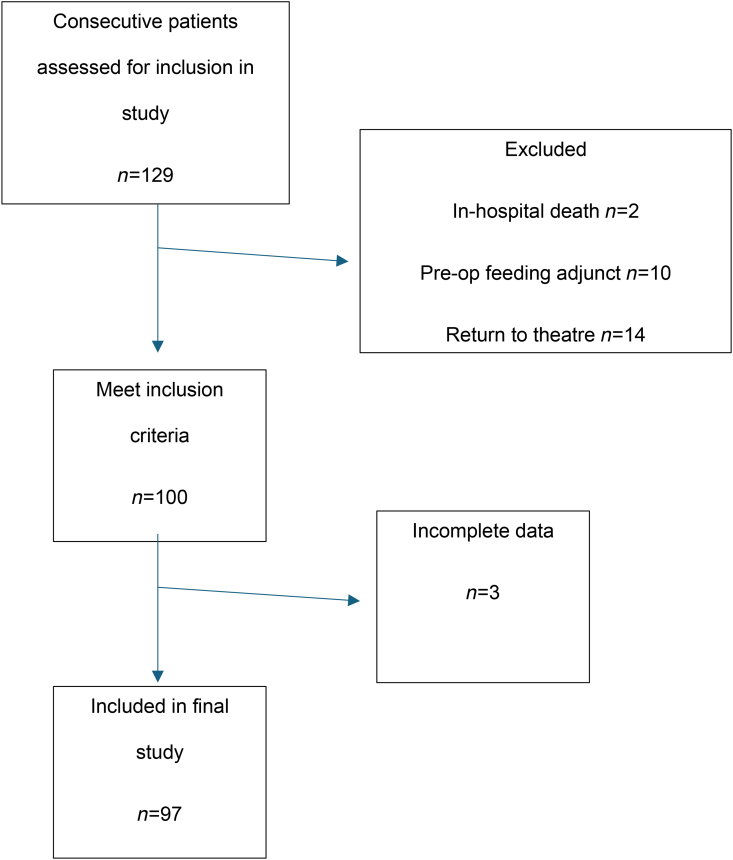
Flowchart of patients included in study

Of the 129 patients screened during the study period, 100 eligible patients were identified; complete data for 97 patients were included for final data analysis (Figure 1).

### Patient demographic and baseline characteristics

Patients were predominantly male, 73/97 (75.3%), with a median age of 70 (range, 47–85 years). Demographic details are presented in Table 1. All included patients had FJ placed at surgery. Only 9/97 (9.3%) of patients required ongoing FJ usage on discharge. There was no significant difference between age, sex, BMI or MUST score between patients who required FJ supplementation on discharge (FJ feed group) and those who did not (no FJ feed group). Patients in the no FJ feed group were more likely to have a Charlson score of 0 (65.9% versus 11.1%); however, this was not statistically significant (*p*=0.075). No patients discharged without FJ feed were subsequently started on FJ feed.

**Table 1:** Patient demographic and clinical characteristics

Variables	All patients (%) *n*=97	Those without FJ feed on discharge (%) *n*=88	Those with FJ feed on discharge (%) *n*=9	*P* value
Sex				
Male	73 (75.3)	67 (76.1)	6 (66.7)	0.679
Age (median, range)	70 (47–85)	70 (47–85)	72 (59–79)	0.761
Charlson comorbidity index				
0	59 (60.8)	58 (65.9)	1 (11.1)	0.075
1	27 (27.8)	22 (25)	5 (55.5)	
2	9 (9.3)	8 (9.1)	1 (11.1)	
≥3	2 (2.1)	0 (0)	2 (22.2)	
BMI at diagnosis (median, range)	27 (17.6–40)	27.1 (17.6–40)	27 (22–34)	0.780
BMI at surgery (median, range)	26.6 (17.2–39)	26.5 (17.2–39)	27 (21–34)	0.120
BMI difference between ‘at diagnosis’ and ‘at surgery’	−0.4	−0.5	0	0.399
MUST score				
0	26 (26.8)	23 (26.1)	3 (33.3)	0.124
1	13 (13.4)	12 (13.6)	1 (11.1)	
2	35 (36.1)	33 (37.5)	2 (22.2)	
3	12 (12.4)	12 (13.6)	0 (0)	
≥4	4 (4.1)	4 (4.5)	0 (0)	
Not documented	7 (7.2)	4 (4.5)	3 (33.3)	

BMI = body mass index; FJ = feeding jejunostomy; MUST = malnutrition universal screening tool

### Surgical outcomes

Surgical outcomes are reported in Table 2. Postoperative complications (by definition, Clavien-Dindo <3 in keeping with inclusion criteria) were seen in 42/97 (43.3%) patients, most commonly respiratory complications. Among those with postoperative complications, four patients required FJ feed upon discharge.

**Table 2:** Surgical outcomes

Variables	*n* (%)
Length of stay in hospital, days	11^a^ (7–43)^b^
Complications	42 (43.3)
Hospital-acquired pneumonia	34 (35.1)
Pleural effusion	12 (12.4)
Pneumothorax	4 (4.1)
Thoracotomy site wound infection	3 (3.1)
Mediastinitis	1 (1)
Sepsis	1 (1)
COVID pneumonitis	1 (1)
Anastomotic leak	1 (1)
Chyle leak	1 (1)
Central line sepsis	1 (1)

^a^Median.

^b^Range.

### FJ outcomes

FJ-related complications (Table 3) were recorded in 18/97 (18.5%) patients, with FJ tube displacement being noted as the most common complications among 7/97 (7.2%) patients, followed by infectious complications. In the excluded group, FJ-related complications were recorded in 6/26 (23.1%) patients. Similarly, FJ tube displacement was recorded as the most frequent complication among 5/26 (19.2%) patients, followed by infectious complications. Clavien-Dindo grading for patients with FJ-related complications who were included in and excluded from the study are demonstrated in Table 3.

**Table 3:** FJ-related complication rate and Clavien-Dindo grading for patients included in and excluded from the study

Variables	Patients included, *n* (%)	Patients excluded, *n* (%)
Total complications	18 (18.5)	6 (23.1)
Tube displacement	7 (7.2)	5 (19.2)
Wound infection	4 (4.1)	1 (3.8)
Leakage	4 (4.1)	0
Pain	3 (3.1)	0
Tube blockage	1 (1)	0
Excoriated skin from jejunostomy stitches	1 (1)	0
Clavien-Dindo grading		
1	7 (7.2)	0 (0)
2	11 (11.3)	6 (23.1)
3a	0 (0)	0 (0)
3b	0 (0)	0 (0)
4	0 (0)	0 (0)
5	0 (0)	0 (0)

FJ = feeding jejunostomy

Among the nine patients who were discharged with a home enteral feeding regimen (Table 4) using FJ, six had multiple comorbidities, including hypertension, diabetes mellitus, heart failure and ischaemic heart disease. All were assessed to be unable to achieve sufficient oral nutrition, generally owing to poor appetite as a result of treatment. All but one patient (who discontinued FJ feeding after 29 days) required long-term feeding exceeding one month postdischarge.

**Table 4:** Individual assessment of patients requiring enteral feed via FJ after discharge

Age	Comorbidities	MUST score	BMI	Length of Stay	Complications
75	HTN, IHD, AF, CKD	0	21	7	None
79	T2DM, IHD, CABG	0	27	8	None
67	T2DM, HTN	1	25	11	Chyle leak
76	HTN, COPD, prostate cancer	n/a	28.5	17	None
67	None	0	25	10	HAP
59	HIV	2	34	11	None
72	HTN, AF, HF, CKD3, hypertrophic cardiomyopathy	n/a	27	10	Pleural effusion
74	HTN, T2DM, IHD, previous bowel and breast cancer	n/a	27	23	COVID pneumonitis
71	IHD	2	22	43	Anastomotic leak (type 1)

AF = atrial fibrillation; BMI = body mass index; CABG = coronary artery bypass grafting; CKD = chronic kidney disease; COPD = chronic obstructive pulmonary disease; FJ = feeding jejunostomy; HAP = hospital-acquired pneumonia; HIV = human immunodeficiency viruses; HTN = hypertension; IHD = ischaemic heart disease; MUST = malnutrition universal screening tool; T2DM = type 2 diabetes mellitus.

Among the ten patients who had preoperative feeding adjunct, five were discharged with FJ feed regimen.

## Discussion

This study reports a low utility of FJ in uncomplicated recovery after oesophagectomy, suggesting a selective, rather than universal, FJ placement strategy may be appropriate. This study reports a low utilisation rate (9.3%) of FJ for home enteral feeding after discharge, but a significant FJ-related complication rate (17.5%). Avoidance of FJ may, therefore, contribute to a reduction in overall postoperative morbidity and re-admission rates, as well as reducing patient discomfort relating to FJ.

These findings are supported by existing publications that have assessed the need for FJ placement in all patients after oesophagectomy. Turner *et al* published a recent nationwide cohort study on FJ tube utilisation in patients undergoing oesophagectomy, and concluded a significant downward trend of FJ utilisation across nine years (60.0% in 2010 versus 41.7% in 2019, *p*<0.01). They highlighted a significantly higher all-cause morbidity rate (40.4% versus 35.5%, *p*=0.01) and notable wound complications (17.0% versus 14.1%, *p*=0.02) in those with FJ compared with those without.^19^ Similarly, in a retrospective study of 262 patients with FJ after oesophagectomy, Couper *et al* demonstrated that only 19% of patients relied upon FJ for enteral feed 20 days after surgery and 81% of patients’ FJs were not useful at time of discharge.^13^ Fenton *et al* also questioned the value of FJ routine use as 77% of the patients’ FJs were not used for enteral feed for one month after oesophagectomy.^14^ Some researchers have even questioned the utility of FJ overall; whereas some studies have emphasised the positive nutritional value of FJ,^20–22^ others have questioned this. Carroll *et al* proposed that FJ delayed but did not prevent weight lost after oesophagectomy when comparing the differences in relative weight and BMI in groups of patients with or without FJ.^23^ Kroese *et al* studied weight loss in those with and without FJ after minimally invasive oesophagectomy (MIE) and presented no significant difference in weight lost in between the groups at three and six months after the operation.^24^

Optimising nutrition intake after oesophagectomy is of paramount importance. ERAS programmes are now applied more widely to different fields of surgery, with early oral feeding one of the key components. The international, multicentre NUTRIENT II randomised controlled trial evaluated the effect of directly starting oral feed after MIE and demonstrated no increase in severity or incidence of postoperative complications. However, the high anastomotic leak rate in both the intervention and control groups raised questions and concerns.^25^ Alternative methods of nutritional support may include the use of parenteral nutrition (PN), nasojejunal tube (NJ) or direct percutaneous endoscopy jejunostomy (DPEJ). The usage of PN is limited by cost, need for central venous access and associated risk of septic complications. Enteral feed being the preferred route of nutritional support, NJ tubes are technically easy to place, but can be uncomfortable to patients and may dislodge. Agarwal *et al* reported a single-centre randomised trial comparing NJ tube placement and FJ for patients undergoing oesophagectomy, and reported that there were no significant differences in postoperative complication rate and catheter-related complication rate, with the NJ group scoring better on the physical domain quality of life (QoL) questionnaire.^26^ Conversely, a recent prospective randomised trial conducted by Tao *et al* studied the differences in overall complication rates between nasogastric feed and FJ groups after minimally invasive McKeown oesophagectomy, and concluded comparable complications between both groups. QoL scores were better in those who had FJ.^27^ DPEJ is used infrequently in surgical practice, but has been reported to have a high technical success rate in some series.^28^ Studies with longer-term comparative outcomes remain sparse.

In pursuing a more selective strategy of FJ placement, we initially intended to conduct a regression analysis to attempt to predict those patients who would be most likely to benefit from FJ. The lack of any significant association between patient or disease factors and need for FJ prevented this. These data would appear to suggest that feeding adjuncts should continue to be considered in a subset of patients, with the most frail or malnourished, or those with significant social or psychological challenges being the most likely to need FJ, in addition to those already dependent on adjuncts in the preoperative phase, but that absolute criteria should be avoided in favour of a multidisciplinary assessment.

The findings reported here should be interpreted in the context of their limitations. The exclusion of patients who suffered major complications from this study reflects the fact that, in patients who suffer major complications, and as such are subject to prolonged hospitalisation, enteral feed via FJ is our routinely preferred route of nutrition. These patients are discharged routinely with ongoing FJ feed and therefore do not answer the question of whether FJ can be avoided in this cohort. As a single-centre case series, findings may be limited in their generalisability to other populations. However, overall surgical outcomes approximate those published in larger national and international case series.^17,29^ Longer term outcomes were not recorded as part of this dataset, so we were unable to capture whether the short-term avoidance of FJ affected longer term nutrition, weight or subsequent interventions such as adjuvant therapy. While long-term survival was not assessed for the same reasons, neither were long-term complications of FJ such as obstruction, volvulus or persistent fistula captured. Another limitation lies in the choice of utilising the Freka enteral feeding tube in all cases, which could have an impact on tube displacement rate compared with other tube types, despite limited evidence on this topic.

## Conclusion

Current practice is gradually evolving away from routine FJ placement due to concerns over poor patient tolerance and associated complications. The data presented here report a low rate of postoperative usage of FJ in a routine placement strategy, which is outweighed by a relatively high rate of FJ-associated complications. This suggests that a limited or selective FJ placement strategy would appear to be safe and can potentially improve short-term outcomes by reducing FJ-related complications, without compromising patient safety and nutrition.

## Ethics approval

This study was carried out under the Code of Ethics of the World Medical Association (Declaration of Helsinki). Approval was granted by the Ethics Committee/IRB of Portsmouth Hospitals University NHS Trust.
